# Science-based taxonomy still needs better governance: Response to Thomson et al.

**DOI:** 10.1371/journal.pbio.2005249

**Published:** 2018-03-14

**Authors:** Stephen Thomas Garnett, Les Christidis

**Affiliations:** 1 Research Institute for the Environment and Livelihoods, Charles Darwin University, Darwin NT, Australia; 2 National Marine Science Centre (NMSC), Southern Cross University, Coffs Harbour NSW, Australia

We thank Thompson et al. [1] for engaging in public debate on improving the governance of taxonomy, even if we are not completely in agreement [2]. As it happens, we think most of their arguments, and concerns about our suggestions, are based on a false premise—it is not we who confuse taxonomy and nomenclature—in fact, nomenclatural rules for taxonomy are a superb example of science governing itself to create a globally acceptable process for naming taxa in an orderly manner. However, the suggestion that they can be tweaked to exclude ‘taxonomic vandals’ may do exactly what we have been accused of doing—stifle taxonomic research. We wish neither to prevent mavericks publishing nor inhibit the excellent work done by the many authors who have contributed to Thomson et al.’s paper. Rather, what we wish to stimulate is a constructive discussion of the process of testing taxonomic hypotheses before they are accepted, not before they are published—this is governance of taxonomy, not nomenclature.

Thomson et al.’s suggestion that legislators change their understanding of species to accommodate modern taxonomic thought is unlikely to happen while there is disagreement about species definitions. Instead, even now, lawyers are actively seeking to impose their own definitions of species, as demonstrated by a recent petition for the United States to adopt the Biological Species Concept as the taxonomic standard for the Endangered Species Act [3]. We proposed a process whereby taxonomists themselves could take the initiative, with lawyers providing advice on the wording of definitions that would make them defensible from just the sort of legal approach now the subject of the petition. Now it seems taxonomists will be advising lawyers on species definitions, not the other way around.

Given the increasing economic impact of taxonomic decisions, one might indeed have expected the Convention on International Trade in Endangered Species (CITES) [4] and International Union for Conservation of Nature (IUCN) [5] to provide a solution, as Thomson et al. suggest. However, both organisations lack a clear governance system for managing taxonomic change in a systematic way. Instead, they follow different taxonomic references for the same groups, with many of the standard texts long out of date. For example, one of the avian texts under CITES dates back to 1975 [6].

In our view, there remain two problems for taxonomic governance outside the realms of nomenclature. The one to which we tried to draw attention, the differing standards for species definition adopted for mammals and birds, may currently be insoluble, the differences too deeply institutionalised and our proposals doomed, at least in the short term. As noted above, the law may take this out of the hands of taxonomists anyway.

The other is a need for legitimised global checklists that conservation authorities can follow. As Thomson et al. note, many such lists are in place or under development, with even the authors of the four competing global bird checklists now in conversation about how to harmonise standards. What is lacking, however, is any global governance structure to give such lists legitimacy. To us, this seems a much more tractable issue than species definitions. There needs only to be a set of criteria for a global list to show it has followed a sufficiently rigorous process in its assembly as well as a body of taxonomists and systematists to say that a list has met those criteria. Such lists could then become the standards for groups such as CITES, IUCN, the Convention for Biological Diversity, and all their attendant agreements and legislation.

## Abbreviations

CITES: Convention on International Trade in Endangered Species
IUCN: International Union for Conservation of Nature
